# A First Approach to Differences in Continuity of Care Perceived by Immigrants and Natives in the Catalan Public Healthcare System 

**DOI:** 10.3390/ijerph10041474

**Published:** 2013-04-08

**Authors:** Marta-Beatriz Aller, Josep Maria Colomé, Sina Waibel, Ingrid Vargas, María Luisa Vázquez

**Affiliations:** 1 Health Policy and Health Services Research Group, Health Policy Research Unit, Consortium for Health Care and Social Services of Catalonia, Barcelona 08022, Spain; E-Mails: doctormia@hotmail.com (J.M.C.); swaibel@consorci.org (S.W.); ivargas@consorci.org (I.V.); mlvazquez@consorci.org (M.L.V.); 2 Ph.D. Programme in Public Health and Methodology of Biomedical Research, Department of Pediatrics, Obstetrics and Gynecology, and Preventive Medicine, Universitat Autònoma de Barcelona (UAB), Barcelona 08193, Spain

**Keywords:** emigrants and immigrants, continuity of patient care, quality of healthcare, health care surveys

## Abstract

*Objective*: To compare immigrants’ and natives’ perceptions of relational, managerial and informational continuity of care and to explore the influence of the length of stay on immigrants’ perceptions of continuity. *Methods*: Cross-sectional study based on a survey of a random sample of 1,500 patients, of which 22% (331) were immigrants. The study area was made up by three healthcare areas of the Catalan healthcare system. To collect data, the CCAENA questionnaire was applied. Multivariate logistic regression models were conducted. *Results*: Like natives, immigrants perceive high levels of managerial continuity (88.5%) and relational continuity with primary and secondary care physicians (86.7 and 81.8%), and lower levels of informational continuity (59.1%). There were no statistically significant differences in managerial and informational continuity between immigrants and natives. However, immigrants perceive a worse relational continuity with primary care physicians in terms of trust, communication and clinical responsibility. Conversely, immigrants perceive higher relational continuity with secondary care physicians in terms of effective communication and clinical responsibility. *Discussion*: Similar managerial and informational continuity perceptions seem to point towards a similar treatment of patients, regardless of their immigrant status. However, differences in relational continuity highlight the need for improvements in professionals’ skills in treating immigrants’ patients.

## 1. Introduction

The profile of the population attended to by the Catalan healthcare services has changed as a consequence of the remarkable increase in immigrant population: while in 2000 foreign born residents accounted for 2.9% of the Catalonian population, by 2010 these figures had reached 15.9% [1]. The three majority groups come from Central and South America (32.5%), Europe (30.3%) and North-Africa and the Maghreb (20.6%) [1]. Until 2012, all immigrants living in Spain were entitled to healthcare coverage under equal conditions as natives, irrespective of their administrative status [2]. Hence, healthcare services faced the challenge of having to adapt to respond to needs and the cultural specificities of this population. 

The Spanish National Health System is financed by taxes and decentralized into regional health services, with universal coverage and free access at point of delivery [3]. Healthcare provision is organised into primary and secondary care, in which primary care is the gatekeeper and secondary care is responsible for the treatment of severe conditions. In order to ensure continuity of care, citizens are assigned to a primary care team that coordinates their care along the care continuum [3,4]. In the Spanish region of Catalonia, the healthcare system is characterized by a split of the financing and provision functions. Healthcare provision is the responsibility of a number of contracted providers; this diversity implies a greater risk of care fragmentation.

As in other countries, in Spain and Catalonia continuity of care has been garnering more attention in the last few years due to the increase in healthcare complexity, high specialization and the involvement of a number of services, as well as the increase in patients with chronic diseases and multiple conditions [5,6]. A number of strategies have been promoted for guaranteeing seamless interfaces; among others, the introduction of integrated healthcare networks, whose ultimate objectives are to improve continuity of care and global efficiency by means of enhancing coordination of care [7,8]. According to the Reid *et al*. conceptual framework, continuity of care is defined as “the degree to which patients experience care over time as coherent and linked” [9,10] and is the result, from the patients’ perspective, of a combination of adequate access to care, good interpersonal skills, good information flow and uptake between professionals and organizations, and good care coordination between professionals to maintain care consistency [9]. Three types of continuity are identified [9,10]: (a) informational: patients’ perceptions of the availability, use and interpretation of information on past events in order to provide care which is appropriate to their current circumstances, (b) managerial: patients’ perceptions of receiving different services in a coordinated, complementary and unduplicated way, and (c) relational: patients’ perceptions of an ongoing, therapeutic relationship with one or more providers. Increased relational continuity has been associated with improved patient outcomes and satisfaction; however, the association between information or management continuity and outcomes is uncertain [11]. 

Previous studies indicate that some population groups are more likely to perceive low levels of continuity of care than others, such as younger patients [12,13,14,15,16,17]. However, the effect of the immigration status on care continuity has scarcely been explored, despite immigrants being particularly vulnerable when care is not provided in a seamless manner between healthcare settings [18]. Indeed, the few existing studies which compare immigrant and native perceptions aimed to analyse certain attributes of relational continuity at the primary care level [19,20,21], concluding that immigrants tend to perceive a worse relationship with professionals than natives in terms of communication [19] and perceived clinical responsibility [20,21]. Some quantitative [21,22] and qualitative research [23,24,25] has explored factors that may influence some attributes of relational continuity [21,22,24,25]. These factors can be grouped into three categories: (1) related to immigrants: insufficient knowledge of the healthcare system, different language, styles of communication and expectations [21,23]; (2) related to physicians, such as prejudices and misunderstandings of immigrants’ views of symptoms and illness [22,23,24,25]; and (3) related to the healthcare services, including the additional time required for consultation with immigrants or the impossibility of accessing their medical histories in other countries [23,25]. The influence of other factors on the perception of continuity of care such as the length of stay in the host country or healthcare systems in their country of origin, has not been explored, although it is known that these factors influence other aspects of care, such as access to healthcare [26,27,28,29]. However, it is important when studying immigrant population to consider that this is a very diverse group with respect to culture and ethnic features, historical roots, and practices concerning health [30].

The relevant percentage of immigrants living in Catalonia and the goal of universal coverage, together with the diversity of health providers, make the Catalan healthcare system an interesting scenario for the comparative analysis of native and immigrant continuity of care perceptions. Research questions were as follows: are the perceptions of continuity of care of immigrant patients similar to those of natives? Do the perceptions of these two patient groups tend to converge when the immigrants’ length of stay in Spain increases? The aim of this article is to compare immigrant and native perceptions of informational, managerial and relational continuity of care, and to explore the influence of the length of stay on immigrants’ perceptions of continuity. 

## 2. Methods

A cross-sectional study was carried out by means of a survey of users of the Catalan public healthcare system. Three healthcare areas were selected in order to represent the diversity of providers present in Catalonia. A single provider supplies both primary and secondary care services in Baix Empordà (Serveis de Salut Integrats del Baix Empordà—SSIBE; a public entity under private law) and in Girona (Institut Català de la Salut—ICS; a public entity under public law). In Ciutat Vella, two entities supply primary care (ICS and Institut de Prestacions d’Assistència Mèdica al Personal Municipal—PAMEM) and a different entity provides secondary care (Parc Salut Mar). The effect of the healthcare area on continuity of care perceptions has been explored elsewhere [16,17]. The registered adult population (18 or over) of the study areas is 74,144 in Baix Empordà, 83,312 in Girona and 99,093 in Ciutat Vella; and immigrants represent 22.1%, 20.6% and 41.2% of this population respectively [1].

### 2.1. Participants

The study population consisted of patients of 18 years of age or over who had received primary and secondary care in the study areas for the same condition in the three months prior to the survey. Sample size was calculated to analyse the model of association between variables at 95% confidence level, to fulfil the de Moivre theorem of expected frequency greater than five and to express the fit and likelihood statistics as a chi-square distribution. The sample size required was approximately 400 patients per healthcare area. The final sample size was 1,500, of which 22% (331) were immigrants.

A simple random sample of patients without replacement was selected from a list of patients that fulfil inclusion criteria. This list was created from records provided by primary care centres and hospitals of the healthcare areas. A list of substitutes which included individuals of the same sex and age group was used to replace any refusals. Patients who had not been attended to by medical professionals or who could not understand or communicate effectively in Spanish or Catalan were excluded.

### 2.2. Data Collection

The Questionnaire of Continuity between Care Levels (CCAENA^©^) questionnaire was applied, which is designed to comprehensively evaluate patients’ experiences of informational, relational and managerial continuity between levels. This tool, previously validated in Spanish and Catalan [31], is divided into two sections: the first reconstructs the care trajectory for a specific episode, and the second, which is the object of this paper, consists of four Likert scales that measure patients’ perceptions of the three types of continuity. Two scales concern relational continuity: the primary and the secondary physician-patient relationship scales, which encompass attributes of trust between provider and patient, sense of clinical responsibility and effective communication. The third scale is related to informational continuity, the information transfer scale, which includes the physician’s knowledge of the patient’s medical history and the supply of timely and adequate information to the patient. The fourth scale refers to managerial continuity, the consistency of care scale, which refers to the coordination between healthcare providers and an adequate sequence of care.

Data were collected by means of face-to-face interviews conducted by trained interviewers from January to May 2010. 

### 2.3. Measures

Explanatory variables: The main explanatory variable was immigration status, defined as being born outside Spain (yes/no). The specific question interviewees were asked was: “Where were you born?” The variable was categorized into three groups according to their length of stay in Spain: short (less than five years), medium (between five and ten years) and long (more than ten years). 

Additional variables were sociodemographic characteristics (sex, age and education level), self-perceived health status and study area. Age was categorized into four groups (18 to 35; 36 to 50; 51 to 65; over 65); educational level into four groups (no education or incomplete primary education; completed primary education; completed secondary education; university education); self-perceived health status into two groups (very good and good; fair, poor and very poor); and study area into three groups (Baix Empordà; Girona; Ciutat Vella).

Outcome measures: Variables that reflected the general perception of continuity were synthetic indexes, computed from the items that constitute the Likert scales (Appendix Table A1). Items had four response options, which varied according to the scale: (1) strongly agree, agree, disagree and strongly disagree, in the relational continuity scales; and (2) always, often, rarely and never, in the informational and managerial continuity scales. 

To estimate continuity indexes, items were scored from 0 to 3 (from strongly disagree/never to strongly agree/always). The simple imputation method was applied based on the mean score of the item, which is considered to be adequate due to the high proportion of complete cases [32]. The second step consisted of summing the scores of each item and dividing them by the highest possible score. In order to simplify the analysis and the presentation of the results, each continuity index was transformed into a dichotomous variable representing (very) high *versus* (very) low perceived levels of care continuity. 

### 2.4. Analysis

A series of logistic regression models were generated in order to evaluate the relationship between variables. Robust covariance adjustments, employing the healthcare area variable, were used to account for correlated observations due to clustering [33]. Percentages and adjusted odds ratios (OR) were calculated for perceived high levels of continuity. The significance level was set at 0.05. 

As differences in perceived relational continuity between natives and immigrants were observed, an additional analysis of these scale items was conducted, which included logistic regression models according to previous specifications. 

Statistical analyses were carried out using Data Analysis and Statistical Software (STATA) Version 11.

### 2.5. Ethical Considerations

The study was conducted in accordance with the current European and Spanish legislation on ethical research. Informed consent was obtained from every interviewee and confidentiality of data was assured through anonymous analysis. The study protocol was approved by the Ethical Committee for Clinical Research Parc Salut Mar (2009/3414/I).

## 3. Results

Interviews were mainly carried out in primary care centres (93.7%), and to a lesser degree in patients’ homes (6.1%) or other locations selected by patients (0.2%). Of the patients contacted, 77.5% refused to take part in the study. There were no statistically significant differences between the final sample and the study population in terms of sex and age. Immigrants represent 16.6%, 17.4% and 29.7% of the sample in Baix Empordà, Girona and Ciutat Vella respectively. Immigrant patients were younger, with higher levels of education, and were healthier than natives: they have a better perceived health status and fewer medical conditions than native patients (Table 1). The 21.9% of immigrants had been in Spain for less than five years, and over half (53.6%) were from Central or South America; 19.1% were from North-Africa and the Maghreb and 16.7% from Europe. 

**Table 1:** Characteristics of the sample.

Characteristics	Natives n (%)	Immigrants n (%)	*p*-value ^a^
Healthcare area			
Baix Empordà	412 (35.2)	82 (24.8)	
Girona	336 (28.7)	71 (21.5)	
Ciutat Vella (Barcelona)	421(36.0)	178 (53.8)	
Sex			
Female	666 (57.0)	184 (55.6)	0.65
Age			
18–35 years	115 (9.8)	119 (36.0)	<0.001
36–50 years	215 (18.4)	133 (40.2)	
51–65 years	335 (28.7)	57 (17.2)	
>65 years	504 (43.1)	22 (6.6)	
Level of education			
No education or incomplete primary education	223 (19.1)	48 (14.6)	<0.001
Completed primary education	326 (27.9)	41 (12.5)	
Completed secondary education	455 (39.0)	160 (48.6)	
University education	164 (14.0)	80 (24.3)	
Self-perceived health status			
Very good, good	528 (45.2)	171 (51.7)	0.04
Fair, poor, very poor	640 (54.8)	160 (48.3)	
Length of stay			
<5 years	-	72 (21.9)	
5 to 10 years	-	150 (45.6)	
>10 years	-	107 (32.5)	
Region of origin			
Central and South America	-	176 (53.3)	
North-Africa and the Maghreb	-	63 (19.1)	
Europe	-	55 (16.7)	
Asia	-	23 (7.0)	
Sub-Saharan Africa and South Central Africa	-	12 (3.6)	
North America	-	1 (0.3)	

^a^ Two-tailed *p*-value from chi-square test.

### 3.1. Perceptions of Continuity of Care

Both natives and immigrants perceived high levels of managerial and relational continuity. However, a large proportion of patients perceived low levels of informational continuity in both populations (Table 2).

**Table 2:** Descriptive analysis and logistic regression models: adjusted relationships between continuity of care perceptions and immigration status according to the length of residence in Spain.

Type of continuity of care	Dimension	Natives (n = 1,169)	Immigrants						
			All	<5 years of residence (n = 72)		5–10 years of residence (n = 150)		>10 years of residence (n = 107)	
		% ^a^	% ^a^	% ^a^	OR (95%IC) ^b^	% ^a^	OR (95%IC)^ b^	% ^a^	OR (95%IC)^ b^
**Informational continuity**	**Transfer of information** (n = 1,448)	74.3	59.1	51.4	1.0 (0.6, 1.7)	55.9	1.0 (0.9, 1.1)	69.3	1.1 (0.8, 1.7)
**Managerial continuity**	**Consistency of care **(n = 1,450)	91.5	88.5	87.0	1.2 (0.3, 4.5)	88.9	1.3 (0.9, 1.8)	88.8	0.9 (0.4, 2.4)
**Relational continuity **	**PC physician-patient relationship **(n = 1,499)	95.6	86.7	83.3	**0.3** (0.1, 0.9)	85.3	**0.4** (0.3, 0.5)	90.6	**0.5 **(0.3, 0.9)
	**SC physician-patient relationship** (n = 1,496)	85.2	81.8	81.9	1.5 (0.8, 2.7)	77.3	1.0 (0.6, 1.9)	88.7	**1.8** (1.4, 2.4)

**^a^** Patients who perceived good or very good continuity;**^b^** Logistic models adjusted for healthcare area, age, sex, level of education, and self-perceived health status. Reference category: natives. Statistically significant ORs are shown in bold.

**Figure 1:**
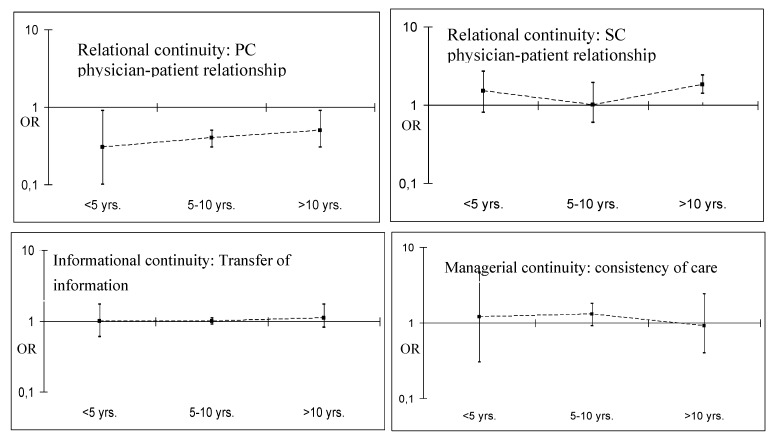
Odds Ratio of adjusted relationships between perceptions of continuity of care and immigration status according to the length of stay in Spain. Logistic models adjusted forhealthcare area, age, sex, level of education, and self-perceived health status.Reference category: natives.

Immigrants tended to perceive worse informational and managerial continuity of care than natives; however, after adjusting for sociodemographic characteristics, self-perceived health status and study area, no significant differences were observed between the two populations (Table 2). With regard to relational continuity, immigrants were less likely than natives to perceive an ongoing relationship with primary care physicians. In contrast, long-term immigrants were more likely to perceive an ongoing relationship with secondary care physicians. There was a tendency among immigrants to rate their relationship with primary care physicians more favourably the longer they had resided in the country. This association was not observed in the other types of continuity (Figure 1). 

### 3.2. Relational Continuity: The Ongoing Relationship between Patients and Physicians

To get more detailed information on differences in relational continuity perceptions’ between immigrants and natives, an additional analysis of the scale items was conducted. Items were grouped according to the attribute they measure: trust in physicians, effective communication and sense of clinical responsibility (Table 3).

*Trust* in physicians’ technical abilities was high among natives and immigrants. However, multivariate analysis revealed that short and medium-term immigrants were less likely than natives to have confidence in primary care physicians’ professional abilities and medium-term immigrants were less likely than natives to feel comfortable when consulting about doubts or concerns. Moreover, long-term immigrants were less likely than natives to recommend their primary care physicians to relatives and friends (Table 3).

Although both immigrants and natives tended to perceive *effective communication* with their physicians, significant differences were observed between the two groups. These differences varied depending on whether items addressed communication with primary or secondary care physicians. On the one hand, natives were more likely to perceive (1) that primary care physicians understand their expectations than medium-term immigrants; (2) that information from primary care physicians is easy to understand than short and long-term immigrants and (3) that primary care physicians give them sufficient information than short and long-term immigrants. On the other hand, natives were less likely to perceive that information from physicians was easy to understand than short and long-term immigrants. 

**Table 3:** Logistic regression models: association of immigration status with items from the physician-patient relationship scales.

Attribute of the physician-patient relationship	Content of items	Level of care	Natives(n = 1,169)	Immigrants					
				>5 years of residence (n = 72)		5–10 years of residence (n = 150)		>10 years of residence (n = 107)	
			% ^a^	% ^a^	OR (95%IC) ^b^	% ^a^	OR (95%IC)^ b^	% ^a^	OR (95%IC)^ b^
**Trust in physicians**	Confidence in the professional ability of physicians	PC	96.7	83.3	**0.3** (0.1, 0.7)	88.0	**0.4** (0.2, 0.6)	91.6	0.4 (0.1, 1.4)
		SC	90.5	79.2	0.7 (0.5, 1.1)	85.9	1.1 (0.6, 1.8)	88.6	1.0 (0.6, 1.9)
	Comfortable consulting about doubts or health problems	PC	95.5	88.9	0.5 (0.2, 1.4)	84.0	**0.3** (0.3, 0.4)	93.4	0.8 (0.6, 1.0)
		SC	85.6	80.6	1.1 (0.3, 3.5)	76.7	0.9 (0.6, 1.3)	84.9	1.3 (1.0, 1.7)
	Recommendation of physicians to relatives and friends if necessary	PC	89.6	70.0	0.4 (0.2, 1.1)	77.1	**0.5 **(0.4, 0.6)	79.8	**0.5** (0.5, 0.7)
		SC	74.1	68.6	1.4 (0.5, 3.9)	72.3	1.5 (0.9, 2.4)	67.3	0.9 (0.7, 1.2)
**Effective communication**	Physicians’ understanding of patient’s explanations	PC	96.9	88.9	0.5 (0.2, 1.0)	89.2	**0.4** (0.3, 0.5)	95.2	0.7 (0.4, 1.3)
		SC	90.0	87.5	1.3 (0.3, 5.1)	89.3	1.6 (0.9, 2.9)	87.6	1.1 (0.8, 1.6)
	Information from physicians is easy to understand	PC	96.6	87.5	**0.1** (0.1, 0.7)	94.0	**0.1** (0.3, 0.9)	94.4	**0.1** (0.3, 0.7)
		SC	86.9	88.9	**1.9** (1.6, 2.2)	84.7	1.2 (0.6, 2.7)	87.7	**1.3** (1.2, 1.5)
	Physicians give sufficient information to patients	PC	87.9	68.0	**0.5** (0.3, 0.8)	73.8	0.6 (0.3, 1.1)	79.0	**0.6 **(0.6, 0.6)
		SC	72.4	61.1	1.1 (0.6, 1.8)	63.1	1.1 (0.8, 1.6)	71.1	1.3 (0.9, 1.7)
**Sense of clinical responsibility **	Sense of clinical responsibility	PC	94.2	76.1	**0.4** (0.1, 0.9)	79.3	**0.3** (0.2, 0.6)	88.3	**0.5 **(0.5, 0.6)
		SC	82.4	72.9	1.1 (0.8, 1.5)	73.5	1.1 (0.6, 1.8)	80.6	**1.3** (1.2, 1.3)

**^s^** Patients that answered “agree” or “totally agree” to items; **^b^** Logistic models adjusted forhealthcare area, age, sex, level of education, and self-perceived health status.Reference category: natives. Statistically significant ORs are shown in bold.Abbreviations: PC: primary care; SC: secondary care.

Finally, most immigrants and natives felt that their physicians had a *sense of clinical responsibility*. While immigrants were less likely than natives to perceive that their primary care physicians feel responsible for them, long-term immigrants were more likely than natives to perceive that secondary care physicians care about them. 

## 4. Discussion

This is the first study to analyse continuity of care perceived by immigrants and natives, and it is exploratory in nature, therefore its results should contribute to guiding future research on the topic. Our main findings indicate that there are no statistically significant differences between immigrant and native populations in perceptions of informational and managerial continuity after adjusting for individual characteristics (such as sex, age or level of education) and study area; however, statistically significant differences were observed in relational continuity. On the one hand, immigrants perceived a worse relationship with primary care physicians than natives in terms of trust, effective communication and clinical responsibility; however, there was a tendency to perceive a better relationship as their length of stay in the country increases. On the other hand, immigrants perceived a better relationship with secondary care physicians than natives in terms of effective communication and clinical responsibility. 

This research was conducted before two important events took place within the Spanish healthcare system: a reduction in the healthcare budget and the implementation of a law which restricts the access of undocumented immigrants to healthcare services [34]. The present study will provide a base-line to analyse the potential impact of these measures on healthcare provision to immigrants. 

### 4.1. Immigrants and Natives Perceive Similar Levels of Informational and Managerial Continuity

Differences observed when comparing crude proportions of immigrant and native perceptions of managerial and informational continuity disappeared after adjusting according to the main variables associated with continuity of care perceptions. This may reflect the different socioeconomic characteristics of immigrants and natives; in fact, immigrants were younger and had higher levels of education than natives – factors associated with worse perceptions of continuity of care [12,13,16]. In addition, almost half the immigrants were from the study area where continuity of care perceptions were lower [16]. Since immigrants constitute a highly heterogeneous group [30], it is possible that some specific groups of immigrants would perceive higher or lower degrees of continuity of care, which need to be explored in further research. 

### 4.2. Worse Perceptions of Relational Continuity with Primary Care Physicians among Immigrants

The higher probability of immigrants perceiving lower levels of relational continuity with primary care physicians is consistent with the results of previous research analysing some aspects of this type of continuity [19,20,21]. The study also shows that there are differences in all attributes of relational continuity with primary-care physicians, *i.e.*, trust, effective communication and clinical responsibility. Immigrants’ characteristics could have an impact on constructing their perceptions, such as different expectations for the type of relationship they have with physicians or different communication styles [21,23]. Different and specific care needs may also affect their expectations and experiences of care [35] and may collaborate to explain the differences observed between the two groups. Causes related to healthcare professionals, such as prejudices and misunderstandings of immigrants’ views, and related to organizations, such as insufficient consultation time, could also explain the differences observed [22,23,24,25]. 

Given the heterogeneity of the immigrant population in aspects such as native language or type of health system in their country of origin [36], it is be expected that immigrants’ perceptions of relational continuity are also highly heterogeneous. In addition, results indicated that their relationship with primary care physicians tends to improve with their length of stay. This finding could be explained by the fact that some factors which negatively affect immigrant perceptions of relational continuity with primary care professionals, such as language proficiency or their expectations and attitude towards professionals, are modified with time. Nevertheless, as mentioned above, although the perception of immigrants improves with the length of stay, their perceptions are always worse than those observed in the native population.

### 4.3. Better Perceptions of Relational Continuity with Secondary Care Physicians among Immigrants

It should be noted that immigrants perceived an ongoing relationship with secondary care physicians to a greater degree than natives, especially when they were asked about their understanding of the information given by secondary care physicians. Since the analysis has been adjusted according to certain variables that may explain the observed differences, such as age and level of education [16], other factors must be the cause of these differences: immigrants could have different expectations with regard to the information given by secondary care physicians, which could affect their assessment of this care element. It is also possible that secondary care physicians make an extra effort to explain clearly with immigrants, which could be reflected in our results. Further research is needed in order to better understand these differences. 

### 4.4. Limitations of the Study

Certain limitations of the study make it difficult to reach any general conclusions. Firstly, 77.5% of patients contacted refused to participate. Although they were replaced by others belonging to the same age group and sex, a non-response bias cannot be ruled out, which could lead to the misrepresentation of certain population characteristics. For example, the distribution of the immigrant population could be biased towards the Latin American population and immigrants who have been in Spain for a long time, since one inclusion criteria was to understand and be able to communicate in Spanish or Catalan. Consequently, it is plausible to assume that the results underestimate potential differences in continuity of care between the immigrant and native population, especially in terms of relational continuity, since available research indicates that patients with a different first language experience a worse relationship with professionals [37,38]. The effect of heterogeneity in the immigration group has not been explored, since the sample size was insufficient to detect differences among immigrant subgroups. This is due to the fact that the study was initially designed to explore organizational and individual factors associated with continuity of care perceptions [16,17] and the results presented here correspond to an additional analysis. Lastly, due to the scarcity of studies analyzing factors associated with continuity of care perceptions, it is possible that not all the variables associated with both immigrant status and continuity of care perceptions have been considered.

## 5. Conclusions

This study has provided the first available evidence of differences between immigrants and natives in their perceptions of the three types of continuity. Results point towards similar perceptions of informational and managerial continuity to those of natives, which seem to indicate that patients receive the same treatment regardless of their immigration status. However, the research also highlights that immigrants perceive lower levels of relational continuity with the primary care physician, thus highlighting the need to improve professionals’ skills in order to improve care for immigrant patients. Furthermore, immigrants perceive higher levels of relational continuity with secondary care professionals than natives. These results highlight the need for further research to identify the underlying causes of the differences observed between immigrant and native populations, as well as to explore immigrant characteristics that may affect their perceptions of continuity of care. Additional in-depth analysis from a qualitative perspective may shed light on the interpretation of these results.

## Appendix

**Table A1:** Items that constitute each Likert scale.

Type of continuity of care	Dimension of continuity of care	Item content
Relational continuity	PC physician-patient relationship	I have confidence in the professional ability of my GP
		I feel comfortable consulting my GP about my doubts or health problems
		I would recommend my GP to my family and friends
		I believe that my GP cares about me
		My GP understands what I tell him/her about my health
		The information my GP gives me is easy to understand
		The information my GP gives me is sufficient
Relational continuity	SC physician-patient relationship	I have confidence in the professional ability of the specialists treating me
		I feel comfortable consulting the specialists about my doubts
		I would recommend my specialists to my friends and family
		I believe that the specialists care about me
		The specialists understand what I tell them about my health
		The information the specialists give me is easy to understand
		The information the specialists give me is sufficient
Informational continuity	Information transfer	I believe that the professionals attending to me know my previous medical history
		My GP is aware of the instructions given to me by the specialist before I explain them to him/her
		The specialist is aware of the instructions given to me by my GP before I explain them to him/her
		After seeing the specialist my GP discusses the visit with me
Managerial continuity	Care coherence	My GP is in agreement with the specialist’s instructions
		The specialist is usually in agreement with my GP’s instructions
		I believe that the care I receive from my GP and the specialist is coordinated

Abbreviations: GP: general practitioner; PC: primary care; SC: secondary care.
